# Evaluation of Alternative Colony Hybridization Methods for Pathogenic Vibrios

**DOI:** 10.3390/foods12071472

**Published:** 2023-03-30

**Authors:** Andrew M. Schwartz, Haley A. Marcotte, Crystal N. Johnson

**Affiliations:** Department of Environmental Sciences, Louisiana State University, Baton Rouge, LA 70803, USA

**Keywords:** vibrio, *tdh*, oyster, probe, hybridization, seafood, pathogens

## Abstract

Vibrios, such as *Vibrio parahaemolyticus*, are naturally occurring halophilic bacteria that are a major cause of foodborne illness. Because of their autochthonous nature, managing vibrio levels in marine and estuarine environments is impossible. Instead, it is crucial to reliably enumerate their abundance to minimize human exposure. One method of achieving this is the direct plating/colony hybridization (DP/CH) method, which has been used to efficiently quantify pathogenic vibrios in oysters and other seafood products. Although successful, the method relies on proprietary resources. We examined alternative approaches, assessed the influence of the reagent suppliers’ source on enumeration accuracy, and made experimental adjustments that maximized efficiency, sensitivity, and specificity. We report here that in-house conjugation via Cell Mosaic is a viable alternative to the previously available sole-source distributor of the alkaline phosphatase-conjugated probes used to enumerate vibrios in oysters. We also report that milk was a viable alternative as a blocking reagent, pH must be eight, an orbital shaker was a viable alternative to a water bath, and narrow polypropylene containers were a viable alternative to Whirl-Pak bags. These modifications will be crucial to scientists enumerating vibrios and other pathogens in food products.

## 1. Introduction

*Vibrio* spp. occur naturally in marine environments. Two species of this genus of bacteria, *V. parahaemolyticus* and *V. vulnificus*, are responsible for most of the reported vibrio-associated illnesses in the United States. *V. parahaemolyticus* is the most common cause of seafood-associated bacterial gastroenteritis with an annual rate of 4500 cases according to the US Centers for Disease Control and Prevention (CDC). Vibrios cause 80,000 illnesses and 100 deaths per year in the United States, according to the CDC (Jones and Oliver). To monitor outbreaks from *Vibrio* spp., experimental methods were created to analyze oysters for the presence of pathogenic vibrios in the 1990s [1]. Direct plating/colony hybridization (DP/CH) is a method widely used to identify and enumerate specific genes in colonies of bacteria [2]. The US Food and Drug Administration (FDA) developed the procedure in 1999 to quantify the presence of pathogenic vibrios in oyster meats to enhance food safety during their raw consumption. This method works by lifting colonies onto Whatman 541 filter paper and then screening the colonies for the gene of interest using an alkaline phosphatase-conjugated oligonucleotide probe. The probe is complementary to the gene of interest and marks the bacterial colonies that contain it by making such colonies appear purple when the chromogenic substrate NBT/BCIP is added.

For over two decades, the DP/CH method has been used in conjunction with real-time quantitative polymerase chain reaction (qPCR) to detect and enumerate pathogenic vibrios. Specifically, we and others have targeted the thermolabile hemolysin (*tlh*) gene found in all *Vibrio parahaemolyticus*, the *vvh* gene found in all *Vibrio vulnificus*, and two pathogenicity factors that are found in *Vibrio parahaemolyticus* [3,4,5,6,7,8,9,10,11]. These are the thermostable direct hemolysin (*tdh*) gene and the *tdh*-related hemolysin (*trh*) gene. Both have been associated with pathogenic and pandemic strains found in human disease.

The DP/CH method developed by the FDA to monitor oysters for pathogenic vibrios using Whatman 541 filters has always relied on a proprietary probe source named DNA Technology A/S in Aarhus, Denmark. The company was acquired in 2013 by Biosearch Technologies and then by LGC Biosearch Technologies in 2015. Unfortunately, LGC Biosearch Technologies discontinued the production of the AP-conjugated probes in 2021. This has rendered researchers no longer able to reliably enumerate pathogenic vibrios in oysters using the DP/CH method because the production details are not publicly available. Thus, we investigated alternatives and present our findings here.

## 2. Materials and Methods

**Figure 1:**
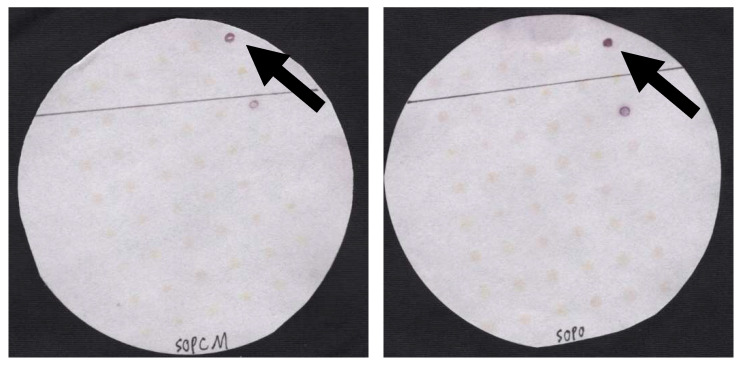
Representative filters probed using AP-*tdh*, created using Cell Mosaic conjugation kit (**left**) and AP-*tdh* purchased from DNA Technology in 2011 (**right**). The positive signals located at the topmost and on the right, as indicated by the arrow, are *V. parahaemolyticus* strain F11-3A (*tdh*+, *trh*+), and the positive signals located two spots below these are *Vibrio parahaemolyticus* strain TX 2103 (*tdh*+, *trh*−). The ImageJ intensity values for the F11-3A signals for the Cell Mosaic AP-*tdh* and for the DNA Technology AP-*tdh* are 116.25 and 96.25, respectively. The intensity values for the negative controls had an average value of 185. The spots corresponding to the F11-3A strain were used for cross-treatment comparisons amongst all filters.

**Creation of control filters.** Control filters were created using established vibrio isolates. The isolates were inoculated into and grown overnight in 10X APW (alkaline peptone water, 1% peptone, 1% NaCl, pH 8.5), and then inoculated onto T_1_N_3_ agar (1% tryptone, 3% NaCl, pH 7.2) using a 48-prong replicator. After overnight incubation at 33 °C, bacterial colonies of approximately 2–3 mm in diameter were lifted and prepared for probing, as described previously [5,6,9,12,13]. Specifically, colonies underwent lifting using Whatman 541 filter paper, cell lysis, and Proteinase K treatment. These filters were subjected to the experimental treatments described below, which target the *tdh* gene in *Vibrio parahaemolyticus*. Two examples of these filters are illustrated in Figure 1.

**Standard DP/CH procedure.** The baseline DP/CH probing process against which all other treatments were compared is as follows. The first step was a 30-min 55 °C incubation in 10 mL of pre-hybridization buffer containing 2% Roche Blocking Reagent (RBR, J. L. Jones, FDA, personal communication), 0.5% polyvinylpyrrolidone (PVP, which increases the hydrophobicity of the Whatman 541 filter paper, thus minimizing non-specific binding of the oligonucleotide), 1% sodium dodecyl sulfate (SDS, which both denatures proteins and increases binding stringency), and 5% fat-free powdered milk (Walmart, Inc., Bentonville, AR, USA), which reduces non-specific binding [14] in a base of 5X SSC.8 (standard saline citrate, 0.75 M NaCl, 0.15 M sodium citrate dihydrate, 0.05 M tris base, pH 8.0). The tris base was used to stabilize the pH. This procedure was carried out in a 55 °C orbital shaker (model # I series 24, New Brunswick Scientific, Edison, NJ, USA). The spent pre-hybridization buffer was decanted and replaced with the hybridization buffer. This was followed by a 1-h 55 °C incubation in 10 mL of hybridization buffer containing 0.5% RBR, 0.5% PVP, 1% SDS, 5% fat-free powdered milk, and the alkaline phosphatase-conjugated *tdh* (AP-*tdh*) probe. The spent hybridization buffer was decanted, and filters underwent two 10-min hot washes in 60–80 mL of pre-heated 2X SSC.8/1% SDS (0.3 M NaCl, 0.06 M sodium citrate dihydrate, 0.05 M tris base, 1% SDS, pH 8.0) at 55 °C. Filters then underwent five 5-min cold rinses in 60–80 mL of 1× SSC.8 (0.15 M NaCl, 0.03 M sodium citrate dihydrate, 0.05 M tris base, pH 8.0) at room temperature. Finally, filters were transferred into a freshly prepared NBT/BCIP solution containing one Ready-to-Go tablet dissolved in 10 mL of deionized water and incubated for color development on an orbital shaker at room temperature for 15–21 h. All incubations were carried out in a 55 °C orbital shaker set to 50 rotations per minute (RPM) with two exceptions: all cold rinses and NBT/BCIP color development were carried out at room temperature and at 100 RPM. The part numbers for the RBR, SDS, PVP, and NBT/BCIP were 11096176001, L5750-1 KG, PVP360-500 G, and 11697471001, respectively (Sigma Aldrich, Aldrich, St. Louis, MO, USA). All probing sessions, or rounds, described throughout this study were carried out in separate containers, and with some exceptions, they were carried out in separate, flat-bottomed, un-textured, air-tight polypropylene containers with 90-mm inside diameters (Ziploc, Twist N’ Loc, size small, San Diego, CA, USA).

**Evaluation of probe source.** To evaluate the effect of the probe source, parallel rounds of probing were carried out using the AP-*tdh* probe created in-house using AP conjugation kits (Cell Mosaic, Woburn, MA, USA) and pre-conjugated AP-*tdh* probes from DNA Technology A/S (Aarhus, Denmark), Biosynthesis (Lewisville, TX, USA), and Click Biosystems (Richardson, TX, USA). The AP-*tdh* probe created in-house used the AP Oligo Conjugation Kit for Single Label with Amine Oligo (Cell Mosaic, Woburn, MA, USA, part number CM53403 × 3). The oligonucleotide used for the kit targeted the thermostable direct hemolysin (*tdh*) gene using the previously established sequence of 5’-XGGTTCTATTCCAAGTAAAATGTATTTG-3’ (IDT DNA, Coralville, IA, USA) in which X was the C6 amino-modifier used to conjugate AP to the oligonucleotide. The probe purification method was HPLC (high-performance liquid chromatography). The average yield that resulted from using the Cell Mosaic kit with the amino-modified oligonucleotide was 56–77 ng/µL, as quantified using a Nanodrop spectrophotometer. The concentration of the Cell Mosaic-conjugated AP-*tdh* used for the treatments described here was 77.2 ng/µL, and 2 µL of the mixture was used per 10 mL of hybridization buffer. The DNA Technology A/S probe was previously purchased in 2011 and had been stored at 4 °C for 12 years, but the Biosynthesis and Click Biosystems AP-*tdh* probes were purchased more recently and had been stored at 4 °C for no more than 2 years. To achieve a final concentration of 5 pmoles per 10 mL of hybridization buffer, we used 12.5 µL of the DNA Technology A/S probe and 10 µL each of the Biosynthesis and Click Biosystems probes. The standard DP/CH procedure was followed as described above.

**Evaluation of blocking reagents.** To evaluate the effects of blocking reagents, pre-hybridization and hybridization buffers served as the experimental treatments. The standard DP/CH procedure described above was modified. The base pre-hybridization buffer contained 0.5% PVP and 0.1% SDS in 5X SSC.8, as above. However, the four main treatments were the addition of RBR, milk powder, both RBR and milk powder, or casein. In cases where RBR was used, the RBR concentrations used in the pre-hybridization buffer and in the hybridization buffer were 2% and 0.5%, respectively (Dr. J. L. Jones, personal communication). In cases where milk powder and casein were used, the final concentrations were 5% and 2%, respectively. The Cell Mosaic AP-*tdh* and the 90-mm polypropylene containers were used across treatments.

**Evaluation of inorganic carbon and pH.** To evaluate the effects of inorganic carbon, similar approaches were used. The impact of inorganic carbon was examined by comparing 1% and 5% NaHCO_3_ in the presence of either 0.5% bovine serum album or 5% milk powder. The base solutions were 5X SSC.8, 2X SSC.8, and 1X SSC.8, as described above. The Cell Mosaic AP-*tdh* and the 90-mm containers were used across treatments. To evaluate the effects of pH, base solutions were set to a pH of 7, and probing was conducted in the presence of 1%, 2%, 5%, and 10% milk powder. The solutions were 5X SSC.7 (0.75 M NaCl, 0.15 M sodium citrate dihydrate, 0.05 M tris base, pH 7.0), 2X SSC.7/1% SDS (0.3 M NaCl, 0.06 M sodium citrate dihydrate, 0.05 M tris base, 1% SDS, pH 7.0), and 1X SSC.7 (0.15 M NaCl, 0.03 M sodium citrate dihydrate, 0.05 M tris base, pH 7.0). The Cell Mosaic AP-*tdh* and the 90-mm containers were used across treatments.

**Evaluation of mechanical parameters.** Similar approaches were used to evaluate the effects of mechanical parameters. In these instances, the conditions were as described above for evaluating the probe source, and the Cell Mosaic-conjugated *tdh* probe was used for every treatment. To evaluate the effect of equipment, hybridization and hot wash steps were carried out in a reciprocal shaking water bath (model # 2870, Thermo Fisher Scientific, Marietta, OH, USA) or in an orbital shaker (model # I series 24, New Brunswick Scientific, Edison, NJ, USA). To evaluate the effect of hybridization vessels, hybridization steps were carried out in Whirl-Pak bags and in two types of polypropylene containers with diameters of 90 mm and 111 mm, respectively.

**Development and analysis of probed filters.** All filters described above were developed in NBT/BCIP at 50–100 RPM at room temperature for 15–21 h, after which they were rinsed three times with 10 mL of deionized water per filter for 10 min per rinse. Filters were dried overnight, scanned individually using an Epson V39 flatbed scanner, and analyzed by ImageJ, a software provided by the National Institutes of Health (NIH, Bethesda, MD, USA). Specifically, images of scanned filters were zoomed to 600%. A circle of approximately one-fourth the size of the F11-3A colony was dragged over the colony and used for the creation of the ImageJ histogram. The mean intensity and RGB (red, green, and blue) values measured the NBT/BCIP precipitate. Each round of probing took approximately 3 hours, and each round was carried out on separate days in separate containers, with one exception: in some figures below, Round 3 represented three filters probed at the same time in the same container. All rounds of probing were paired with the baseline standard described above. Statistical analyses consisted of paired t-tests and were carried out using Microsoft Excel. In cases where a round of probing included three filters at a time, the mean of the three was used to compare to Rounds 1 and 2. Vertical axes were presented in reverse to illustrate the inverse relationship between signal intensity and ImageJ values: higher signal intensities on the probed filters produced lower ImageJ intensity values.

## 3. Results

The use of the Cell Mosaic kit to conjugate AP to the amine-modified *tdh* oligonucleotide as an alternative to the probe previously available from DNA Technology was more effective than the purchase of AP-conjugated oligonucleotides at this time. All probe results reported here were specific; non-specific signals were minimal (Figure 1).

The intensity values as measured by ImageJ were used for the *Vibrio parahaemolyticus* positive control strain F11-3A (*tdh*+, *trh*+) [10] for comparison across treatments (Figure 1). A second positive control strain, TX 2103 (*tdh*+, *trh*−), was also included. Negative controls used in this project included other *Vibrio* spp., including AQ 4037 (*tdh*−, *trh*+) and FIHES (*tdh*−, *trh*−) [10]. These strains originated from the US FDA Gulf Coast Seafood Laboratory in Dauphin Island, AL, USA and were kindly provided by Dr. Jessica L. Jones. Other strains used as negative controls originated from environmental samples from coastal Louisiana oysters. We used real-time qPCR [10] and Sanger sequencing of their 16S rRNA genes [15] to identify them as *tdh*-negative *Vibrio* spp. (Table 1). The mean ImageJ intensity value for the negative controls in this study was 185.

**Figure 2:**
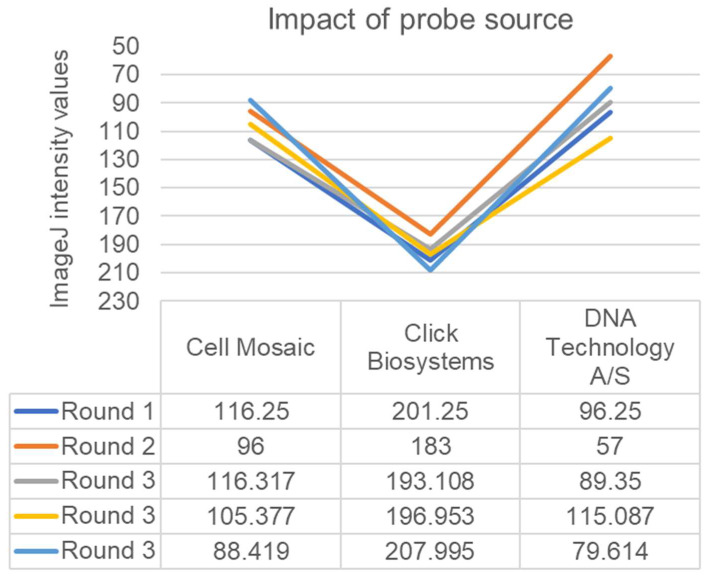
ImageJ intensity values for AP-*tdh* probes purchased from three different sources. Signals resulting from Cell Mosaic and the previous DNA Technology A/S probes were both significantly lower (and thus signals were stronger) than those produced in our hands at this time by the Click Biosystems probes (*p* < 0.05), and they were not significantly different from each other (*p* > 0.05).

When assessing the impact of the probe source, we compared the recently prepared AP-conjugated *tdh* probe resulting from the use of the Cell Mosaic kit to the AP-conjugated *tdh* probe originally purchased in 2011 from DNA Technology and stored at 4 °C for 12 years. The results from the old DNA Technology probe were still superior to the new Cell Mosaic probe (Figure 2). Results from the AP-*tdh* purchased from Biosynthesis remained below the lower limit of detection in our hands, and the results from the AP-*tdh* purchased from Click Biosystems were not reproducible at this time using the conditions tested in our lab. Notably, the AP activity in both of these probes remained very high when added directly to the NBT/BCIP; thus, the signal reductions were associated with the hybridization steps and not the loss of AP effectiveness.

**Figure 3:**
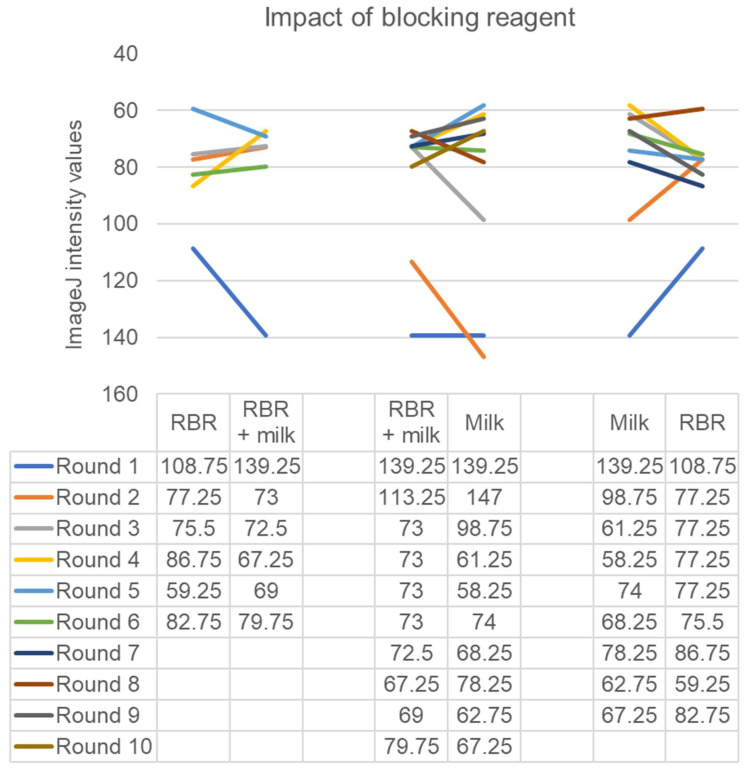
ImageJ intensity values for AP-*tdh* probes paired by blocking conditions. Base hybridization buffers contained 5X SSC.8, 0.5% PVP, and 1% SDS as a base. To this base were added RBR, RBR + 5% milk (RBR + milk), or 5% milk alone (Milk).

When assessing the impacts of blocking conditions, we compared Roche Blocking Reagent (RBR) with milk and casein (Figure 3). Comparing the use of RBR with or without milk to the use of milk alone yielded no statistically significant differences (*p* > 0.05). However, comparisons between milk alone and RBR alone demonstrated that milk alone yielded superior results in six out of nine probing sessions (Figure 3, *p* > 0.05).

The use of casein failed to replicate the use of milk alone (data not shown). Alternative milk sources were also examined (*n* = 1), including fresh Kleinpeter skim milk (Baton Rouge, LA, USA), canned evaporated Carnation milk with 2% milkfat (Los Angeles, CA, USA), and boxed Parmalat milk with 2% milkfat (Wallington, NJ, USA). These were all diluted to the equivalent of a final concentration of 5% milk powder (1:5, 1:10, and 1:5, respectively). These yielded similarly strong results with ImageJ intensity values of 61.25, 58.25, and 74, respectively.

We examined other factors that might impact future results. Probe results using a tris-stabilized pH of 7 instead of 8 yielded comparatively weak signals (mean intensity value of 125.4, *n* = 5). The introduction of 1% or 5% NaHCO_3_ in hybridization buffers failed to strengthen the signal (data not shown).

**Figure 4:**
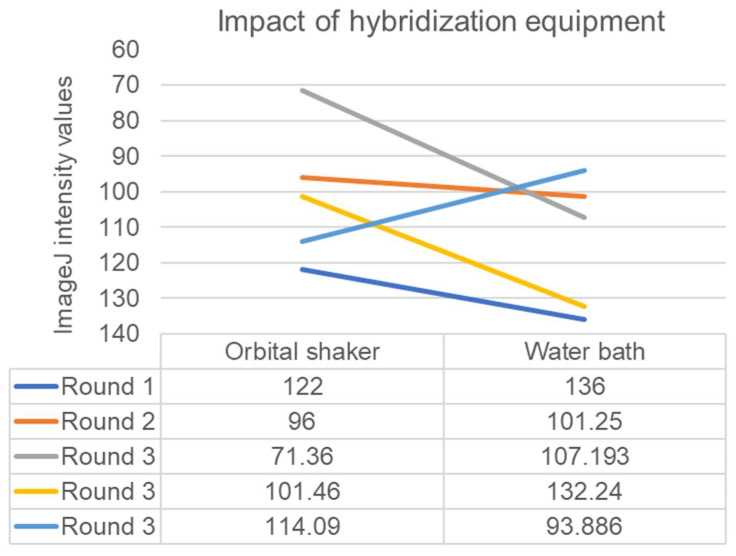
ImageJ intensity values for hybridization steps in orbital shaker versus reciprocal shaking water bath. Signal intensities resulting from the use of an orbital shaker were stronger than those from using a reciprocal shaking water bath in two out of three rounds of probing (*p* = 0.07).

Signal intensities resulting from the use of a heated orbital shaker were stronger than those from using a heated reciprocal shaking water bath in two out of three probing sessions (Figure 4, *p* = 0.07). Shaking speed in the orbital shaker did not appear to impact signal intensity (intensity values of 94.75 with shaking at 50 RPM versus 100 with shaking at 100 RPM, *n* = 1).

**Figure 5:**
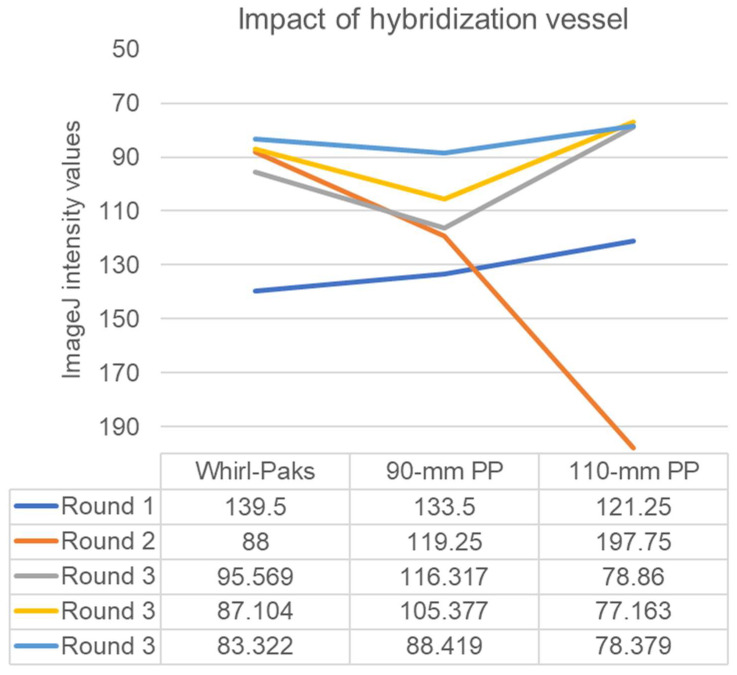
ImageJ intensity values for hybridization steps in heated orbital shaker versus reciprocal shaking heated water bath. Whirl-Pak bags yielded results superior to the 90-mm containers in two out of three probing sessions (*p* > 0.05).

Comparisons amongst container types yielded interesting results (Figure 5). We compared the results of probing using Whirl-Pak bags, which held the 85-mm round filters in a 100 mm × 100 mm square enclosure, to the results of probing using a 90-mm inside diameter round polypropylene containers and 111-mm inside diameter round polypropylene containers in a heated orbital shaker. Whirl-Pak bags yielded results superior to the 90-mm containers in two out of three probing sessions, but overall there were no significant differences (*p* > 0.05). Interestingly, Round 2 illustrated the problem with using the 111-mm container. The single filter was not sufficiently exposed to the probe suspended in the hybridization buffer, visible as inconsistent coloring on the filter, and this problem would be further compounded with the FDA-accepted standard probing of five filters per round.

The thickness of Whatman 541 filters is approximately 0.155 mm per filter (Cytiva Life Sciences, Marlborough, MA, USA). The working areas of the Whirl-Pak bags and the 90-mm and 111-mm containers were 10,000 mm^2^, 6359 mm^2^, and 9672 mm^2^, respectively. Thus, solving for height in volume = area × height indicates they had hybridization buffer heights of 1 mm, 1.57 mm, and 1.03 mm, respectively, when stationary. Thus, the use of the 90-mm round polypropylene container yielded a higher exposure of filters to the probe, a reduced risk of air bubbles and pipet contamination, and a faster return to the high hybridization temperatures compared to the wider 111-mm jar and the 100 × 100 mm Whirl-Pak bags.

Other conditions tested include: 0.5–2% bovine serum albumin (BSA), Denhardt’s buffer [16], Church buffer [17], phosphate buffered saline (PBS), tris-buffered saline, 0–0.75% SDS, Tris-EDTA at pH of 8, 0.5M EDTA at pH of 8, 50% formamide, 0.2 mg/mL salmon sperm DNA, 0.05% *v*/*v* tween-20, 0.08% triton X-100, 0.1% ficoll, 5% dextran sulfate, 1–10% milk powder, increased NaCl concentrations, increased probe concentrations, increased and decreased SSC concentrations, hybridization temperatures of 37 to 60 °C, extended hybridization and hot wash steps times, tap water, and carbonated water. All of these conditions yielded inferior results that included weak signals and/or unacceptably high levels of non-specificity.

## 4. Discussion

Vibrios are naturally occurring halophilic bacteria that can accumulate in oysters when they filter water [18]. Thus, exposure to vibrios is a frequent cause of seafood-borne illnesses [19,20,21]. Vibrio densities vary with environmental conditions. We and others have demonstrated that vibrio concentrations are higher during warm months and lower during colder months [5,12,22]. Their densities are also related to salinity, chlorophyll, turbidity, and other factors [5,6,20,23,24,25,26,27,28]. Because vibrios naturally occur in marine and estuarine environments, the most effective way to minimize infections is to minimize exposure to these bacteria when their concentrations are high. Exposure prevention and assessment of seafood safety are possible if these pathogens and the environmental conditions that favor their increased densities can be reliably measured.

The enumeration of vibrios, particularly pathogenic *Vibrio parahaemolyticus*, in environmental food sources such as oysters destined for raw consumption is crucial to accurately assess the risk of, and thus minimize, infectious disease exposure in humans. A large proportion of microbes found in the environment, which includes human pathogens, are not culturable, and their presence is only known because of the detection of 16S rRNA signals. Reasons for this include the inability to culture them using typical growth media and the survival of the bacteria in the viable but nonculturable (VBNC) state [29,30,31].

However, one benefit of culture-based methods is accessibility. The DP/CH method is simple and allows for the enumeration of vibrios globally in laboratories in developing countries where Illumina sequencing is not as accessible. Another benefit of culture-based methods is the isolation and curation of isolates for further characterization and phylogenetic comparison to well-established global isolates, thus drawing phylogenetic connections between pandemic strains and those isolated from the environment [6]. The use of DP/CH also makes it possible to assess trends across multiple decades and institutions. One major caveat to the DP/CH method has been the reliance on the use of proprietary probes such as those previously available from DNA Technology A/S. In a time when corporate uncertainty is a factor in any scientific research endeavor, it is imperative to proactively identify alternative approaches and methodological flexibilities. The study described here provides a crucial step in the identification of alternative approaches to DP/CH enumeration of vibrios and other foodborne pathogens.

Indeed, DP/CH enumeration has been reliably used for over two decades and has successfully informed critical risk assessments and studies of environmental pathogens. The US FDA developed the current and nearly universally used DP/CH method [1,32,33]. This method was quickly adopted and used to develop the now-standard risk assessment for *Vibrio parahaemolyticus* [34,35]. The strength, integrity, accessibility, and optimization of this method continue to this day to inform the FDA BAM (Bacteriological Analytical Manual) [36,37], which hundreds of scientists use annually to enumerate vibrios and other pathogenic bacteria to ensure food safety [38,39,40,41,42,43]. The method is also included in the vibrio risk assessment developed by the FAO and WHO (Food and Agriculture Organization of the United Nations and the World Health Organization) [44,45].

Here, we report the identification of a viable alternative to pre-conjugated oligonucleotide probes: in-house conjugation using the Cell Mosaic kit. The process required approximately four hours of hands-on time during the course of two days. The equipment required was conventional, including a microcentrifuge (refrigeration beneficial but not required), a vortex, micropipettes, a 4 °C refrigerator, and a 37 °C incubator. The cost of the kit plus the amine-modified, HPLC-purified oligonucleotide was similar to the original AP-*tdh* from DNA Technology A/S.

Our finding that previously purchased and stored AP-*tdh* probe was more effective than freshly purchased and prepared AP-*tdh* probe was interesting. DNA is very stable, but the AP enzyme is generally labile. However, the ability of the expired enzyme to act on the NBT/BCIP substrate remained intact for 12 years. The nature of the original conjugation between the *tdh* oligonucleotide and the AP enzyme by the scientists at DNA Technology A/S is unknown, but the original product was shipped in 0.1% sodium azide, 1 mg/mL acetylated BSA, 20 mM Tris-HCl, pH 7.5, and 500 mM NaCl. The nature of the Cell Mosaic AP conjugation step, however, is more transparent. The process starts with a C6-amine-modified oligonucleotide, converts it to a thiol-oligonucleotide, and ends with a reaction between the thiol-oligonucleotide and a maleimide-activated alkaline phosphatase enzyme originating from calf intestine. The formulation of the final elution is also provided by the manufacturer: 400 μL of 50 mM Tris buffer, pH 8.0, 1 M NaCl, 1 mM MgCl_2_, and 0.1 mM ZnCl_2_. The AP-*tdh* probe from Click Biosystems that originally yielded successful probing results was shipped and stored in a buffer of 25 mM Tris-HCl, 1 mM MgCl_2_, and 0.1 mM ZnCl_2_, pH 7.6. The AP-*tdh* probe from BioSynthesis was shipped and stored in a buffer of 10 mM HEPES buffer, 150 mM NaCl, pH 7.4. Both the Click Biosystems and Biosynthesis probes were conjugated using Click chemistry, the mechanism of which is proprietary.

The other proprietary facet of this study was the use of the Roche Blocking Reagent, which has been successfully used to minimize non-specific binding in DNA-based hybridization reactions [36,46]. We report here that commonly available milk yields similarly strong and specific signals, if not more so. The RBR product is proprietary, but the material safety data sheet lists casein as one of the ingredients. Unfortunately, we were unable to reproduce the effect of RBR when using casein as a blocking reagent. Future studies will examine whey and other biochemical components, but the discovery that milk powder was as effective as RBR in increasing stringency as a blocking agent was a success. In addition, milk is often locally available, and the milk powder used for the majority of the hybridizations in this study averages US$0.011 per gram while the RBR cost US$3.54 per gram and was frequently back-ordered.

DNA hybridization has been used for several decades [47]. In general, a custom oligonucleotide fragment is generated with a sequence that is complementary to a desired gene target. The oligonucleotide is modified with an indicator that can be detected after hybridization with the target. If the indicator is a radioisotope such as ^13^C, a molecule such as biotin or digoxigenin, a fluorophore such as fluorescein, or an enzyme such as horseradish peroxidase, then the hybridization to the target can be detected using radiograph film, a labelled antibody to the molecule, a fluorescence microscope, or a substrate for the molecule, respectively. Here, we used an enzyme, alkaline phosphatase (AP). AP acts on a chromogenic substrate, NBT-BCIP, to produce a blue precipitate that can be seen with the naked eye, thus removing the need for specialized equipment.

In hybridization methods, efforts are made to increase the likelihood that the labelled oligonucleotide only hybridizes with the target, including hybridizing at high temperatures, the addition of a chemical such as PVP to increase the hydrophobicity of the filter paper, the addition of a detergent such as SDS to maximize access to intracellular targets [48], and the addition of a blocking reagent that increases the stringency of the interaction between the oligonucleotide and the target by reducing non-specific binding. In the past, blocking reagents have included BSA, ficoll, and salmon sperm DNA, in addition to the two reagents we successfully used here, milk and RBR. Our success at hybridization specificity with milk compared to other blocking reagents tested is insightful because it increases the accessibility of the method on a global scale and minimizes dependence on expensive and frequently back-ordered reagents.

In this study, we also demonstrated the success of using a polypropylene container with a narrower diameter, foregoing the Whirl-Pak bag and the shaking water bath altogether. The use of these containers, which were essentially airtight cups, made it possible to probe large numbers of filters at a time without a cooling effect on the pre-hybridization and hybridization buffers. Specifically, we were able to quickly remove the lids from these containers, add the respective probe, and return the containers to the heated orbital shaker. This was highly efficient compared to Whirl-Pak bags, which are plagued by air bubbles and prone to close in on and thus contaminate pipetting devices. There was one caveat, however. During the addition of the pre-hybridization buffer, it was imperative to meticulously coat each side of every filter and only add them one at a time. This step required some adjustment and was the rate-limiting step in the probing process in our hands. Thus, when probing five filters plus one control strip, we used 11 mL of pre-hybridization buffer but kept the volume of the hybridization buffer at 10 mL to maintain the probe-to-diluent ratio. In this study, we used the Ziploc Twist N’ Loc, size small. However, an alternative container can be used instead as long as the diameter is similar and there is no protrusion in the middle of the polypropylene container. Unsurprisingly, the large diameter container yielded inferior results because the smaller diameter allowed for more exposure of the filters to the probe and a more efficient use of space.

Another discovery was the difficulty in standardizing color development across treatments. This challenge applies across research groups as well. In addition, photographic documentation of probed Whatman 541 filters is notoriously inaccurate: both DSLR and mobile cameras dull the positive controls and artificially amplify the negative controls. The use of ImageJ was beneficial in that it provided an intensity value that could be used to compare signals. However, when analyzing samples, such as colony lifts from oyster samples, it can sometimes be difficult to discern a positive signal from background non-specificity. Thus we developed a graphic based on the RGB (red, green, and blue) values for the positive controls used in this study, and these are illustrated in the graphical abstract.

The major limitation of this study was the inability to compare more AP-conjugated *tdh* probes. Unfortunately, corporate sources were rare, we were only successful in acquiring the AP-*tdh* from two companies, and we were unable to produce consistent results at this time. Thankfully, Cell Mosaic provided an AP-conjugation kit that yielded consistent results. Future studies will examine digoxigenin- or biotin-conjugated oligonucleotides, as these are more readily available from biotech companies but are not yet standardized for the DP/CH procedure, which uses Whatman 541 filters. Future studies will also include the targeting of other genes such as *trh* (*tdh*-related hemolysin) and targeting of other bacteria, including pathogenic *Vibrio vulnificus* [49,50], fecal coliforms, and antibiotic-resistant bacteria [51,52]. We also aim to reproduce the successful Click BioSystems and BioSynthesis results we achieved in 2021.

The second limitation is that different operators can indeed generate different results. This is particularly true if different reagents and equipment are used. For example, it is important that if a polypropylene container is used, it must have a flat bottom. The hybridization buffer must be kept as hot as possible once the probe is added to minimize non-specific binding; thus, this step must proceed quickly. Air bubbles must be prevented. Times and temperatures must be held constant between probing sessions. We observed variation in results even amongst filters probed at the same time in the same containers. For example, the data spreads in Round 3 in each of Figure 2, Figure 4 and Figure 5 are wide despite identical conditions. Fortunately, real oyster samples only require an assessment of positive or negative, and this variation becomes less relevant with the appropriate negative controls in place. Thus, to minimize operator-based variability, it is crucial to include control strips with every probing batch of five full-size filters, as described previously [32]. It may also be beneficial to use ImageJ, Matlab, or similar software for the establishment of cutoff points, as illustrated in the graphical abstract. As supplementary information [Supplementary Materials, S1], we submit a more detailed protocol of the parameters we use for this 3-h process.

In summary, we found that (1) oligonucleotide probes generated in-house were specific and sensitive for DP/CH detection of *tdh*, (2) milk, regardless of source and fat content, was at least as good as, if not better than, Roche Blocking Reagent, (3) the 90-mm diameter polypropylene container was as effective as the standard Whirl-Pak bag for the hybridization process, and (4) the orbital shaker is a viable alternative to the water bath. The goal of our study was to maximize the reproducibility, sensitivity, specificity, efficiency, and affordability of the DP/CH method. We also aimed to maximize accessibility for others to increase the surveillance of oysters destined for raw consumption in areas that have not previously used this monitoring approach.

## Figures and Tables

**Table 1:** 16S rRNA gene sequences and taxonomic identifications of isolates used in this study.

Isolate Name	*tlh/tdh/trh/vvh*	16S rRNA Gene Sequence
AMS001	*tlh*+	ACTCCTACGGGAGGCAGCAGTGGGGAATATTGCACAATGGGCGCAAGACCTGATGCAGCCATGCCGCGTGTGTGAAGAAGGCCTTCGGGTTGTAAAGCACTTTCAGTCGTGAGGAAGGTAGTGTAGTTAATAGCTGCATTATTTGACGTTATCGACAGAAGAAGCACCGGCTAACTCCGTGCCAGCAGCCGCGGTAAT
AMS002	*tlh*+	ACTCCTACGGGAGGCAGCAGTGGGGAATATTGCACAATGGGCGCAAGCCTGATGCAGCCATGCCGCGTGTGTGAAGAAGGCCTTCGGGTTGTAAAGCACTTTCAGTCGTGAGGAAGGTAGTGTAGTTAATAGCTGCATTATTTGACGTTAGCGACAGAAGAAGCACCGGCTAACTCCGTGCCAGCAGCCGCGGTAAT
AMS003	*tlh*+	ACTCCTACGGGAGGCAGCAGTGGGGAATATTGCACAATGGGCGCAAGCCTGATGCAGCCATGCCGCGTGTGTGAAGAAGGCCTTCGGGTTGTAAAGCACTTTCAGTCGTGAGGAAGGTAGTGTAGTTAATAGCTGCATTATTTGACGTTATGCTGACAGAAGAAGCACCGGCTAACTCCGTGCCAGCAGCCGCGGTAAT
AMS004	*tlh*+	ACTCCTACGGGAGGCAGCAGTGGGGAATATTGCACAATGGGCGCAAGCCTGATGCAGCCATGCCGCGTGTGTGAAGAAGGCCTTCGGGTTGTAAAGCACTTTCANGTCGTGAGGAAGGTAGTGTAGTTAATAGCTGCATTATTTGACGTTATGCTGACAGAAGAAGCACCGGCTAACTCCGTGCCAGCAGCCGCGGTAAT
AMS005	*tlh*+	ACTCCTACGGGAGGCAGCAGTGGGGAATATTGCACAATGGGCGCAAGCCTGATGCAGCCATGCCGCGTGTGTGAAGAAGGCCTTCGGGTTGTAAAGCACTTTCAGTCGTGAGGAAGGNAGTGTAGTTAATAGCTGCATTATTTGACGTTATCGACAGAAGAAGCACCGGCTAACTCCGTGCCAGCAGCCGCGGTAAT
AMS006	None	ACTCCTACGGGAGGCAGCAGTGGGGAATATTGCACAATGGGGGAAACCCTGATGCAGCCATGCCGCGTGTGTGAAGAAGGCCTTCGGGTTGTAAAGCACTTTCAGTCGTGAGGAAGGCATATGCGTTAATAGCGCATGTGTTTGACGTTATGCTGACAGAAGAAGCACCGGCTAACTCCGTGCCAGCAGCCGCGGTAAT
AMS007	None	ACTCCTACGGGAGGCAGCAGTGGGGAATATTGCACAATGGGCGCAAGCCTGATGCAGCCATGCCGCGTGTGTGAAGAAGGCCTTCGGGTTGTAAAGCACTTTCAGTCGTGAGGAAGGTGGTGTAGTTAATAGCTGCATTACTTGACGTTATGCGACAGAAGAAGCACCGGCTAACTCCGTGCCAGCAGCCGCGGTAAT
AMS008	None	ACTCCTACGGGAGGCAGCAGTGGGGAATATTGCACAATGGGGGAAACCCTGATGCAGCCATGCCGCGTGTGTGAAGAAGGCCTTCGGGTTGTAAAGCACTTTCAGTCGTGAGGAAGGCATATGCGTTAATAGCGCATGTGTTTGACGTTAGCGACAGAAGAAGCACCGGCTAACTCCGTGCCAGCAGCCGCGGTAAT
AMS009	*trh*+	ACTCCTACGGGAGGCAGCAGTGGGGAATATTGCACAATGGGCGCAACGCCTGATGCAGCCATGCCGCGTGTGTGAAGAAGGCCTTCGGGTTGTAAAGCACTTTCAGTCGTGAGGAAGGTAGTGTAGTTAATAGCTGCATTATTTGACGTTATGCGACAGAAGAAGCACCGGCTAACTCCGTGCCAGCAGCCGCGGTAAT
AMS010	*tlh*+	ACTCCTACGGGAGGCAGCAGTGGGGAATATTGCACAATGGGCGCAAGCCTGATGCAGCCATGCCGCGTGTGTGAAGAAGGCCTTCGGGTTGTAAAGCACTTTCAGTCGTGAGGAAGGTAGTGTAGTTAATAGCTGCATTATTTGACGTTAGCGACAGAAGAAGCACCGGCTAACTCCGTGCCAGCAGCCGCGGTAAT
AMS011	*tlh*+	ACTCCTACGGGAGGCAGCAGTGGGGAATATTGCACAATGGGCGCAAGACCTGATGCAGCCATGCCGCGTGTGTGAAGAAGGCCTTCGGGTTGTAAAGCACTTTCAGTCGTGAGGAAGGNNGTGTAGTTAATAGCTGCATTATTTGACGTTATCGACAGAAGAAGCACCGGCTAACTCCGTGCCAGCAGCCGCGGTAAT
Haley	None	ACTCCTACGGGAGGCAGCAGTGGGGAATATTGCACAATGGGCGCAACGCCTGATGCAGCCATGCCGCGTGTATGAAGAAGGCCTTCGGGTTGTAAAGTACTTTCAGTCGTGAGGAAGGGGGTNTCGTTAATAGCNGTATTCTTTGACGTTATCGACAGAAGAAGCACCGGCTAACTCCGTGCCAGCAGCCGCGGTAAT
AMS013	*tlh*+	ACTCCTACGGGAGGCAGCAGTGGGGAATATTGCACAATGGGCGCAAGCCTGATGCAGCCATGCCGCGTGTGTGAAGAAGGCCTTCGGGTTGTAAAGCACTTTCAGTCGTGAGGAAGGTAGTGTAGTTAATAGCTGCATTATTTGACGTTATGCGACAGAAGAAGCACCGGCTAACTCCGTGCCAGCAGCCGCGGTAAT
AMS014	None	ACTCCTACGGGAGGCAGCAGTGGGGAATATTGCACAATGGGCGCAAGCCTGATGCAGCCATGCCGCGTGTGTGAAGAAGGCCTTCGGGTTGTAAAGCACTTTCAGTCGTGAGGAAGGTAGTGTAGTTAATAGCTGCATTATTTGACGTTATGCGACAGAAGAAGCACCGGCTAACTCCGTGCCAGCAGCCGCGGTAAT
AMS015	*tlh*+	ACTCCTACGGGAGGCAGCAGTGGGGAATATTGCACAATGGGCGCAAGCCTGATGCAGCCATGCCGCGTGTGTGAAGAAGGCCTTCGGGTTGTAAAGCACTTTCAGTCGTGAGGAAGGNNGTGTAGTTAATAGCTGCATTATTTGACGTTATGCGACAGAAGAAGCACCGGCTAACTCCGTGCCAGCAGCCGCGGTAAT

Representative samples were analyzed by multiplex qPCR for detection of *tlh*, *tdh*, *trh*, and *vvh* and by Sanger sequencing.

## Data Availability

All data generated from this project are provided within the context of the full text of this manuscript.

## Supplementary Materials

The following supporting information can be downloaded at: https://www.mdpi.com/article/10.3390/foods12071472/s1, Information S1: Probing protocol.
