# Supplementary material for: Evaluation of Alternative Colony Hybridization Methods for Pathogenic Vibrios
Source: Foods. 2023 Mar 30;12(7):1472. doi: 10.3390/foods12071472 (PMC10093671; doi:10.3390/foods12071472)
Supplement: Supplementary file 1 [file foods-12-01472-s001.zip › foods-2244684-supplementary.pdf]

Supplemental Information for Schwartz, et al.

To increase accessibility and understanding of the DP/CH (direct plating/colony hybridization) protocol, we submit our detailed in-house probing protocol written by undergraduate researchers Haley A. Marcotte and Andrew M. Schwartz.

Probing protocol for the Johnson Lab

Reagents (that are not temperature- or time-sensitive):

***When making reagents, add each component in the order that it is written.

20X SSC (Store at room temperature, RT, and in glass bottles only):

Final volumes:	1000 mL	2000 mL
NaCl (grams)	175.4	350.8
Sodium citrate dihydrate (grams)	88.2	176.4
DI (deionized) water (mL)	800	1600

Adjust pH to 7, q.s. to final volumes.

5X SSC.8 (Store at RT and in glass bottles only):

Final volumes:	1000 mL	2000 mL
20X SSC (mL)	250	500
DI water (mL)	750	1500
Tris base (*not Tris HCl*) (grams)	6.057	12.114

Adjust to pH of 8 using strong hydrochloric acid, e.g., 6 M HCl.

2X SSC.8 / 1% SDS (Store at RT and in glass bottles only):

Final volumes (mL):	1000mL	2000mL	3000mL	4000 mL
20X SSC (mL)	100	200	300	400
DI water (mL)	850	1700	2550	3400
Tris base (grams)	6.057	12.114	18.171	24.228
Adjust to pH of 8 using 6 M HCl.				
Add 20% SDS (mL)	50	100	150	200

1X SSC.8 (store at RT, in glass bottles or plastic carboys):

Final volumes (mL):	1000mL	2000mL	3000mL	4000 mL
20X SSC (mL)	50	100	150	200
DI water (mL)	950	1900	2850	3800
Tris base (grams)	6.057	12.114	18.171	24.228

Adjust to pH of 8 using 6 M HCl.

Reagents (that ARE temperature- or time-sensitive):
***When making reagents, add each component in the order that it is written, and use round glass bottles.

Pre-hybridization solution (store up to 1 week at 4°C, and use glass bottles only):
Label bottle “pre-hyb” with date and add stir bar.

**5X SSC.8 (mL)**	**100**	**200**	**300**
Polyvinylpyrrolidone (grams)	0.5	1.0	1.5
**Roche Blocking Reagent (grams)**	**2**	**4**	**6**
SDS (grams)	1	2	3
Milk powder (grams)	5	10	15

Place bottles containing reagents on stir plate, heat and stir at 100°C until everything is dissolved. If using pre-made / refrigerated solution, place on un-heated stir plate, then set temperature to 100°C, then stir at high speed (~350-700 RPM, depending on the size of the stir bar, enough to create a vortex in the bottle) for ~30 minutes or until no longer cool to touch. Also, can pre-heat in other heating equipment such as orbital shaker or water bath then stir on plate for 10 minutes.

Hybridization solution (store up to 1 week at 4°C, and use glass bottles only):
Label bottle “hyb” with date and add stir bar.

**5X SSC.8 (mL)**	**100**	**200**	**300**
Polyvinylpyrrolidone (grams)	0.5	1.0	1.5
**Roche Blocking Reagent (grams)**	**0.5**	**1.0**	**1.5**
SDS (grams)	1	2	3
Milk powder (grams)	5	10	15

Follow same instructions as pre-hyb solution above.

Preparation Checklist:

Select up to 5 filters and 1 control strip that have been treated with Proteinase K according to the established FDA protocol.

Heat orbital shaker to 55°C and move pre-hyb, hyb, and 2X SSC.8 / 1% SDS to orbital shaker.

Check for solutions containing precipitated SDS, especially if room is cold.

- If precipitate is found, shake bottle containing 2XSSC.8 / 1% SDS and place into 55°C orbital shaker.
- Do not continue until precipitate has dissolved.

Once heated at least 15 minutes, put pre-hyb and hyb solution bottles on 55°C stir plate and stir at ~700RPM for 10 minutes.

Have large beaker nearby on workbench for decanting spent solutions to work quickly and keep solutions hot. Also keep a working bottle of 1X SSC.8 nearby as the carboy is slow.

Never probe different gene targets during the same probing session because of increased risk of cross-contamination.

Prepare 90-mm round polypropylene (PP) containers, e.g., Ziploc Twist N' Loc, size small.

- Have twice as many Ziplocs as probing groups because NBT/BCIP will be prepared in a fresh Ziploc.
- Before each re-use, use 70% ethanol and a Kim-wipe to remove any residual NBT/BCIP from all Ziplocs, then rinse with DI water to remove ethanol.

Probing:

1. Pre-hybridization steps:
    a. Put 10-11 mL of 55°C pre-hyb solution into Ziploc using serological pipet.
    b. Add filters one at a time, making sure each one gets saturated and there are no air bubbles. It helps to lean the Ziploc to pool the solution, place each filter into the pool, and slowly submerge the filter.
    c. As more filters are added, use each subsequent filter to "paint" the previously added filter, i.e., use the incoming filter to spread the pre-hyb solution over the top surface of each filter where the colonies are embedded.
    d. **There must be absolutely no dry spots, i.e., there must be a layer of pre-hyb solution between every filter.**
    e. Use the control filter to do the final "paint" step. Work quickly to keep the pre-hyb as hot (and thus fluid) as possible.
    f. Place Ziplocs into 55°C orbital shaker, and shake at 50 RPM for 30 minutes.
2. Hybridization steps:
    a. Several minutes before pre-hyb timer goes off, move probe cryo-box from refrigerator to work bench, set micropipet to 2 µL, and insert 10-mL serological pipet into pipet aid.
    b. When timer goes off, move all Ziplocs and hyb solution from orbital shaker to workbench.
    c. Remove first lid, decant pre-hyb (not a lot of liquid will come out), and leave lid off to the side. Repeat until all Ziplocs are lidless.
    d. The following steps must proceed quickly, one Ziploc at a time, to prevent non-specific binding of the probe to non-target DNA:

        Add 10 mL of 55°C hyb solution to the first Ziploc using serological pipet.
        Add 2 µL probe.
        Swirl to mix.
        Twist lid on tightly.
        Place into orbital shaker.
        Repeat for the next Ziploc.

    e. Shake at 50 RPM for 1 hour.
    f. Place pre-hyb and hyb solutions in refrigerator; leave 2X SSC.8 / 1% SDS hot wash solution heating.
3. Hot Washes:
    a. When timer goes off, move all Ziplocs and 2X SSC/SDS from orbital shaker and place on bench. Remove first lid, decant hyb solution, and leave lid off to the side.

        Tip: If air bubbles or residual solutions are under the filters, slowly rotate the wrist while decanting to remove.

    b. Repeat until all Ziplocs are lidless.
    c. Add ~60-80 mL of 55°C hot wash solution to each, which creates a coverage of ~1 cm of solution over the filters.
    d. Tighten all lids and move Ziplocs and bottle of hot wash solution to orbital shaker.
    e. Shake at 50 RPM for 10 minutes at 55°C.
    f. Decant and hot wash a 2nd time.
    g. During the hot washes, add 10 mL DI water and 1 NBT/BCIP tablet per container to the unused set of Ziplocs.

   h. Place NBT/BCIP Ziplocs onto RT orbital shaker and cover with a black cloth to ensure no light exposure.
4. Cold Rinses:
   a. Remove hot washes from orbital shaker, and remove all Ziploc lids. Decant hot wash.
   b. Pour ~60-80 mL of RT 1X SSC.8 into each Ziploc, and rinse at RT at ~100 RPM for 5 minutes.
   c. Repeat for a total of 5 cold rinses.
5. NBTBCIP incubation
   a. After the final cold rinse, use forceps to move filters *one* at a time into NBT/BCIP solution, using the pooling method used above for the pre-hyb step. Make sure no bubbles stay under the filters.
   b. Shake obscured from light at 50 RPM for 15-21 hours.
6. Next-day rinses
   a. Decant spent NBT/BCIP and pour in generous amount of DI water so that all filters are liberally covered. Close all lids.
   b. Place on RT orbital shaker at ~100 RPM for ten minutes, then discard.
   c. Repeat two more times for a total of 3 rinses.
   d. Place filters cells-side up on clean paper towels to dry.
   e. Allow to dry overnight before placing into a data organization binder.

Reagents list (reagent, part number, source)

1. Sodium dodecyl sulfate (SDS), L5750-1KG, Sigma
2. Polyvinylpyrrolidone (PVP), PVP360-500G, Sigma
3. Roche Blocking Reagent (RBR), 11096176001, Sigma
4. NBT/BCIP, 11697471001, Sigma
5. Fat-free powdered milk, 10415475, Walmart
6. Sodium citrate dihydrate, S279-500, Fisher
7. Tris base, BP152-500, Fisher
8. NaCl, BP358-212, Fisher

Alternatives and adaptations for other researchers that would likely yield equivalent results:

1. Roche Blocking Reagent may be treated as optional, depending on study, consistency, access, and background signals in negative controls.
2. Hybridization solution and probe can be pre-mixed when probing in large batches as long as the mix is kept heated.
3. Ziploc containers are not required. Any 90-mm diameter container with a flat bottom and a tight lid would suffice, including Fisher part number 02-891E.
4. A 55°C reciprocating water bath may be used instead of a heated orbital shaker.
5. The 55°C hybridization temperature may be adjusted depending on the probe sequence.
6. Tris-HCl may be used in lieu of tris base if NaOH is used to adjust the pH of solutions.
7. Other milk types are options for blocking solutions, including 1-2% fat milk, canned evaporated (but not sweetened condensed) milk, and boxed milk.
8. Whirl-Pak bags may be used in lieu of polypropylene containers.
